# Vital role of chest CT in diagnosis of coronavirus disease 2019 (COVID-19)

**DOI:** 10.22088/cjim.11.3.244

**Published:** 2020-05

**Authors:** Zeinab Mohseni Afshar, Soheil Ebrahimpour, Mostafa Javanian, VeneelaKrishna Rekha Vasigala, Jila Masrour-Roudsari, Arefeh Babazadeh

**Affiliations:** 1 Clinical Research Development Center, Imam Reza Hospital, Kermanshah University of Medical Sciences, Kermanshah, Iran; 2 Infectious Diseases and Tropical Medicine Research Center, Health Research Institute, Babol University of Medical Sciences, Babol, Iran; 3 Rangaraya Medical College, NTR University of Health Sciences, Kakinada, India; 4 Student Research Committee, Babol University of Medical Sciences, Babol, Iran

**Keywords:** COVID-19, Chest computed tomography (CT) scan, Reverse transcription polymerase chain reaction (RT-PCR), Ground-glass opacities

## Abstract

In December 2019, a new virus called coronavirus disease 2019 (COVID-19) causing severe acute respiratory syndrome emerged in Wuhan, China, and rapidly spread to other areas of China and other regions of the world. Since it was a discovery, COVID-19 has spread to several countries and to this date, affecting about 2,329,651 people and caused about 160,721 deaths. Since most COVID-19 infected cases were diagnosed with pneumonia and characteristic chest computed tomography (CT) scan patterns, radiological examinations have become an important tool in early diagnosis. Nowadays, CT findings combined with normal blood cells (WBCs), lymphopenia and a history of epidemiological exposure have been used as criteria for clinical diagnosis of COVID-19. It is noteworthy that reverse transcription polymerase chain reaction (RT-PCR) test is still gold standard for the diagnosis. This review focuses on role of chest CT in the clinical evaluation of disease progression and more accurate diagnosis.

Coronavirus is an enveloped host specific RNA virus, and can infect humans as well as a variety of animals. Four of the coronaviruses (229E, OC43, NL63, and HKU1) cause mild upper respiratory tract infections (common cold), whereas three other zoonotic coronaviruses include the severe acute respiratory syndrome coronavirus (SARS-CoV), the Middle East respiratory syndrome coronavirus (MERS-CoV), and coronavirus disease 2019 (COVID-19) which resulted in atypical pneumonia (1-5). COVID-19 caused approximately 2 million confirmed cases and also 160,721 deaths globally until April 18, 2020 (6). Most studies have informed the genome of COVID-19 has about 90% nucleotide identity with bat SARS-like-CoVZXC21 and about 80% with that of human SARS-CoV (7, 8). In reality, information on epidemiology, pathogenesis, clinical signs and radiologic features of the disease is incomplete. As a result, the World Health Organization (WHO) issued case definitions for the diagnosis of suspected and probable cases of COVID-19 (9). According to WHO, a suspected patient is a case with acute respiratory illness (fever and at least one sign/symptom of respiratory disease such as cough, shortness of breath), with no other etiology that completely explains the clinical symptomatology or with a history of travel to an area reporting local transmission of COVID-19 during 14 days prior to symptom onset. Also, the case has been in contact with a probable or confirmed COVID19 case in the last 2 weeks prior to onset of signs and symptoms. A probable case is a suspect patient whose test result for COVID-19 is inconclusive. 

The mortality rate of COVID-19 can be reduced by timely diagnosis of infection particularly in higher risk cases of progressing into critically ill patients, and administering intensive care treatment to these patients (10, 11). Herein, among different methods of diagnosis of infection, we discuss several imaging findings in this new alarming viral infection. It seems that the radiological examination is of great importance in the early detection of COVID-19. 

The imaging results vary with the patient’s age, comorbidities, immunity condition, stage of infection at the time of scanning, and drug interventions. The imaging features of COVID-19 lesions are important in this aspect that they show: (1) dominant distribution (mostly subpleural, along the bronchial vascular bundles); (2) quantity (frequently more than three or more lesions, occasional single or double lesions); (3) shape (patchy, large block, nodular, lumpy, honeycomblike or grid-like, cord-like, etc.); (4) density (mainly uneven, a paving stones-like change mixed with ground glass density and interlobular septal thickening, consolidation and thickened bronchial wall, etc.); and (5) concomitant signs vary (air-bronchogram, rare pleural effusion and mediastinal lymph nodal enlargement, etc.) (12, 13). Usually, the first imaging in a COVID-19 suspected case is a simple chest radiography. Chest x ray most commonly reveals characteristic multiple small patchy infiltrations, progressing to large ground-glass opacities (GGOs) which are usually bilateral (14). GGO is defined as hazy increased lung attenuation with preservation of bronchial and vascular margins, and consolidation is defined as opacification with obscuration of margins of vessels and airway walls. Interstitial changes are often visible, which are prominent in the lung periphery. Gradually pulmonary consolidation becomes evident and sometimes pleural effusion occurs. It is important to note that the chest radiograph may reveal normal findings in the early stage of disease and it is not sensitive for the detection of GGO, hence, it was not recommended as a first-line test for COVID-19 (15). A normal chest CT scan does not exclude the diagnosis of COVID-19 infection.

Sensitivity of the thoracic computed tomography (CT) scan is more than x-ray in identifying viral pneumonia. So, it can be more helpful in the earlier stages of pulmonary involvement. GGO is seen as an increase in attenuation of parenchyma that appears in interstitial and alveolar processes with preservation of the bronchial and vascular margins, while consolidation is an area of opacification obscuring the margins of bronchi and vasculature, without significant loss of lung volume (16). Imaging findings on CT scan include unilateral, focal GGO and segmental consolidation, especially in the subpleural regions that tend to progress to bilateral consolidation in hospitalized patients (17). 

In severely infected intensive care unit (ICU) admitted patients, multiple lobar lesions may be present in both lungs (18). In general, ICU- admitted patients are more likely to have larger areas of bilateral consolidation on CT scans, whereas non-ICU patients with milder illness are more likely to have GGO and small areas of consolidation. These patterns are indicative of extensive interstitial and alveolar lung injury. Other findings observed include patchy densities or infiltrates, crazy-paving pattern of interlobular septa, linear involvement, bilateral hilar infiltration, segmented opacities and even pneumothorax (19). Interestingly, atypical findings have been reported in different COVID-19 case reports, such as halo sign, pulmonary nodules, pleural effusions, lymphadenopathy, reticular pattern, scattered opacities etc, while a substantial proportion of patients had clear lungs without any consolidation or scarring. Some of the aforementioned CT findings can be noticed in adenovirus infection (17). However, tree-in-bud signs, masses, cavitations, and calcifications, often suggestive of bacterial infections are rare findings in COVID-19 infection. Generally, pure multifocal GGO, GGO with reticular and/or interlobular septal thickening, and GGO with consolidation are the key findings. These lesions are distributed peripherally with bilateral involvement of the lungs (20). 

In one retrospective study, the initial plain non-contrast CT scans were assessed for the following characteristics: (a) presence of GGOs, (b) presence of consolidation, (c) number of lobes affected by ground-glass or consolidative opacities, (d) occurrence of a pleural effusion,(e) presence of nodules, (f) degree of lobe involvement, (g) presence of thoracic lymphadenopathy (described as lymph node size of more than 10 mm in short-axis dimension), and (h) presence of underlying lung disease such as emphysema or fibrosis (21). Along with the preceding findings, other abnormalities like cavitation, reticulation, calcification, interlobular septal thickening, airway wall thickening and bronchiectasis were also taken into account. . Depending on the degree of involvement of all the five lung lobes, these patients were classified to have none, minimal, mild, moderate, or severe pulmonary involvement, and then, the decision for their management was made accordingly. Some limitations of this study that might affect the interpretation of the results include, recall bias wherein some subjects were not able to provide the date of appearance of their first symptom, and usage of steroids or antimicrobials that may alter chest findings. The duration between the symptom onset and initial chest CT examination would categorize the subjects as to which phase of illness they are allocated to ie; early, intermediate or late phases(21).

**Changes in lung lesions and pattern of disease progression: **The pulmonary lesions of COVID-19 pneumonia are diverse, varying from GGO in the early stage, progressing to consolidation, and finally resorption with resolution of the disease. However, the greatest severity of these lesions peaked approximately by day 10 after the initial symptom onset (22). 

 The CT imaging reveals five stages based on the time of onset and how the body responds to the virus which include: (1) Ultra-early stage, (2) Early stage, (3) Rapid progression stage, (4) Consolidation stage, and (5) Dissipation stage (23). The typical pattern of progression of CT images is usually unilateral, multifocal, predominantly GGOs in the first week after onset of symptom, that quickly progresses to bilateral, diffuse disease, with a relative decrease in the frequency of ground-glass opacities. Subsequently, there is a decrease in the frequency of ground-glass opacities in the second week after symptom onset, which is dominated by consolidation. (12). 

A reticular pattern indicative of bronchiolectasis and irregular interlobular or septal thickening tend to increase progressively from the second week (12, 14). These findings indicate the appearance of interstitial changes suggesting the development of fibrosis. With the progression of the disease in the third week, consolidation and mixed patterns are seen and the appearance of ground-glass opacities diminished (14). Bronchiolectasis, pleural effusion, and thickening of the adjacent pleura appear at this stage. Another finding discovered in recent studies is that there were more consolidation lesions and less GGO lesions in the elderlies than in the younger ones(24). As consolidation indicates disease progression, such a pathology would demand being more vigilant during the management. Since a considerable number of patients with COVID-19 infection develop acute respiratory distress syndrome (ARDS), we should expect extensive consolidation and ground-glass opacity, which is an archetypal response of acute lung injury (25, 26).

Risk factors of poor prognosis in patients with COVID-19 pneumonia include elderly, male sex, underlying medical conditions, and progressive radiographic deterioration on follow-up CT (26). One research demonstrated that the median time from onset of symptoms to mechanical ventilation was 7 to 14 days and to intensive care unit admission was 8 to 17 days (27). This shows that the radiological evolution of COVID-19 pneumonia is relevant with the clinical course of the disease.

**The differential diagnosis of COVID-19 and comparison of imaging appearance to other pulmonary infections: **CT image findings of COVID-19 infected patients are usually nonspecific and may overlap with infections resulting from streptococcus pneumonia, mycoplasma and chlamydia related pneumonia, SARS-CoV, H7N9 pneumonia, H1N1 virus infection, and avian influenza A (H5N1) (28). Thus, a differential diagnosis is critical for early suspicion of COVID-19 infection in patients with fever and compatible imaging findings, to initiate quarantine efforts and to reduce cross-transmission. 

CT imaging findings in common cold are usually normal. In H1N1 influenza patients, the most common CT findings are airway thickening/dilatation, peribronchial ground glass opacity, centrilobular nodules, and tree-in-bud opacities. The findings frequently involve all lobes and are commonly associated with large and small airways. Also, peripheral consolidation involving the lower lobes is a predominant feature in H1N1 infections (29).

CT scans of SARS-CoV patients commonly show unilateral or bilateral GGO or focal unilateral or bilateral areas of consolidation(30). The infection progresses to bilateral consolidation, mainly involving lower lobes. CT findings of SARS-CoV patients may also present interlobular septal and intralobular septal thickening (31). In MERS-CoV patients, bilateral basilar and subpleural airspace, extensive GGO, and occasional septal thickening and pleural effusions are the main CT findings (32, 33). In mild COVID-19 infections, multifocal patchy GGOs with subpleural distribution is the dominant CT finding. In severe COVID-19 infections, a diffuse heterogenous consolidation with GGO is the common finding. 

A combination of COVID-19 CT findings along with viral distribution in the posterior and the peripheral part of the lungs are important to differentiate COVID-19 from other viral pneumonia infections. In general, H7N9 pneumonia predominantly affects the right lower lung while H1N1 pneumonia and SARS-CoV affect peripheral regions of lungs (24, 34). Lower lobes are usually more affected in COVID-19 infections than the upper lobes in the early phases of COVID-19 illness and radiographic progression is more rapid than SARS associated illness (12). Similar to COVID-19 virus, MERS-CoV virus distribution is predominantly in the posterior and peripheral parts of the lung (17). 

It is suggested that the CT findings for COVID-19 share many similar features with SARS-CoV and MERS-CoV. A crazy-paving pattern (defined as thickened interlobular septa and intralobular lines with superimposed GGO), pleural effusions, absence of pulmonary cavitation, and lymphadenopathy were features in common with SARS-CoV and MERS-CoV (35, 36). However, multifocal involvement is more prominent with COVID-19 infection. In both MERS-CoV and COVID-19 infections, a follow-up CT in the later stages of infection show residual fibrotic changes. In brief, pattern of ground-glass opacities and consolidation, often with a bilateral and peripheral lung distribution is increasingly emerging as the chest CT hallmark of COVID-19 infection.

Epidemiological exposure or close contact with suspected or confirmed cases is an essential clue for the initial diagnosis in a patient with compatible symptoms and signs and graphic evidences. However, for patients with unknown exposure history, typical clinical and imaging findings can indicate a suspected COVID-19 infection. A reverse transcription polymerase chain reaction (RT-PCR) test should be performed in the suspected patients for COVID-19 confirmation. CT findings also have proven to be diagnostic in a number of cases as the sensitivity of chest CT was higher than that of RT-PCR (98% vs 70%) (37). However, some cases have been diagnosed to have initial normal CT scans, suggesting that CT scan alone can reliably exclude this disease, especially in the early stages of infection. In other words, 60% to 90% of cases had initial positive chest CT scans compatible with COVID-19 before the initial positive RT-PCR results (38). Some studies have shown chest CT images have the sensitivity of 80–90% and specificity of 80–95% for detecting the lung lesions in COVID-19 (39). 

In conclusion recognizing the radiological features depending on the course of infection is of paramount importance not only in understanding the disease progression, but also helps to speculate the outcome or any potential complications. Chest CT is an indispensable component in the diagnostic algorithm for patients with suspected infection with COVID-19 outbreak. COVID-19 pneumonia tends to manifest on lung CT scans as bilateral, subpleural, GGO, ill-defined margins, and a slight predominance in the right lower lobe. The imaging characteristics of COVID-19 are non-specific and share similarities with those of SARS-CoV2 and MERS-CoV infections. 

Therefore, CT findings combined with bilateral or peripheral distribution of the virus, normal white blood cells (WBCs), lymphopenia, and a history of epidemical exposure should be considered for suspecting COVID-19 pneumonia cases. In addition, although chest CT findings alone are of little diagnostic value for COVID-19 detection, CT findings are considered as the most important evidence of clinical diagnosis, especially in the early stages of the disease in a number of cases with an initial false-negative RT-PCR screening test.
